# Can current farmland landscapes feed declining steppe birds? Evaluating arthropod abundance for the endangered little bustard (*Tetrax tetrax*) in cereal farmland during the chick‐rearing period: Variations between habitats and localities

**DOI:** 10.1002/ece3.7271

**Published:** 2021-03-02

**Authors:** David González del Portillo, Beatriz Arroyo, Guillermo García Simón, Manuel B. Morales

**Affiliations:** ^1^ Terrestrial Ecology Group (TEG) Department of Ecology, Research Center on Biodiversity and Global Change Autónoma University of Madrid Madrid Spain; ^2^ Instituto de Investigación en Recursos Cinegéticos (IREC) CSIC‐UCLM‐JCCM Ciudad Real Spain

**Keywords:** agriculture intensification, alfalfa, coleopterans, farmland birds, habitat quality, orthopterans

## Abstract

Agriculture intensification threatens farmland bird populations because, among other reasons, it reduces the availability of food resources required to rear their offspring. In our study, we sampled and analyzed total arthropod abundance, biomass and richness, and orthopteran and coleopteran abundance and biomass in different agricultural habitats (alfalfa fields, stubble fields, grazed fields, and field margins) across 4 study localities with different levels of agriculture abandonment–intensification, comparing between areas used and not used by one of the most threatened farmland birds in Europe, the little bustard (*Tetrax tetrax*), during the chick‐rearing season. Field margins were the taxonomically richest habitat, while alfalfa fields presented significantly higher total arthropod abundance and biomass than other habitats. All arthropod variables were the highest in the localities with clear conservation‐focused agrarian management, and the lowest in the most intensive one. Areas used by little bustards had higher orthopteran and coleopteran abundance and biomass than nonused areas, except for coleopteran biomass in grazed fields. These results highlight the relevance of these arthropods for the species, the importance of dry alfalfa fields as food reservoirs in this critical time of year, the food scarcity in sites where agrarian management disregards farmland bird conservation, and the role of stubbles as providers of food resources during the chick‐rearing season in areas used by the species. The adequate management of alfalfa fields and stubbles to provide those key resources seems crucial to improve little bustard breeding success.

## Graphical abstract

We compare invertebrate abundance, biomass, and richness in four different habitats in areas used and not used by little bustards (*Tetrax tetrax*) and across three sites of different farming intensity. Results highlight that traditional farmland management aimed at bird conservation leads to higher food availability and that little bustards favor those areas.

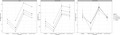

## 1 INTRODUCTION

Over the last 50 years, agricultural management has changed due to the intensification of farming techniques (Chamberlain et al., 2000; Fuller et al., 1995; Sanderson et al., 2005; Santos & Suárez, 2005; Siriwardena et al., 2000). The mosaic of different crops and plots at different stages of the agrarian cycle typical of extensive farmland favored biodiversity and food web interactions (Galbraith, 1988; Östman et al., 2001). One of the most evident results of intensification is the loss of such heterogeneity (Benton et al., 2003; Emmerson et al., 2016; Zamora et al., 2007), which has led to the general decline of farmland biodiversity (Emmerson et al., 2016; Stoate et al., 2009), including plants, invertebrates, and vertebrates (Bas et al., 2009; Benton et al., 2003; Geiger et al., 2010; Sotherton & Self, 2000). A good example is provided by farmland birds in Europe, which have strongly declined across the continent in the last three decades (Donald et al., 2006; Eurostat, 2020).

Intensive agricultural practices include early harvesting, the use of silage systems, the application of agrochemicals, and the reduction of nonproductive, semi‐natural areas such as fallows, hedgerows, and field boundaries, and their ultimate end is to increase crop productivity (Grigg, 1989; O’Connor & Shrubb, 1986; Stoate, 1996; Whittingham et al., 2006). These practices, aided by the mechanization of agricultural works, are at the root of bird farmland population declines, as they are associated with the loss of food resources (Brickle et al., 2000; Campbell et al., 1997; Evans et al., 1997; Potts, 1986), the reduction of nesting areas (Chamberlain et al., 2000; Wilson et al., 1997), or increased mortality (Crick et al., 1994; Green, 1995).

**FIGURE 1:**
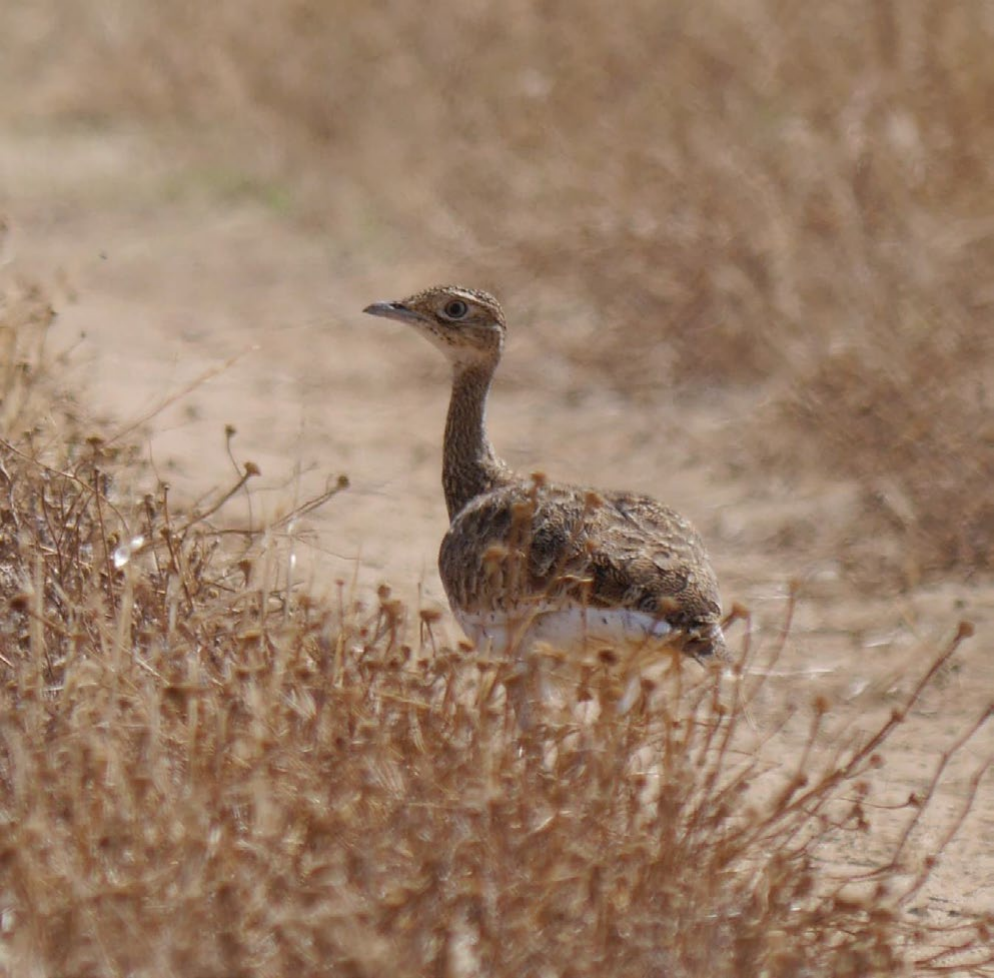
Photograph of a little bustard (young male) taken during censuses

One of the farmland bird species most negatively affected by agriculture intensification in Europe is the little bustard *Tetrax tetrax* (Figure 1). This steppe bird was widely distributed across the Palearctic, from Morocco to West China, in the past, but has suffered a sharp decline over the last decades (Goriup, 1994; Morales & Bretagnolle, unpublished data). The Iberian Peninsula is the species’ stronghold in the western Palearctic (García de la Morena et al., 2018, Morales & Bretagnolle, unpublished data). Nowadays, the little bustard inhabits mainly pseudosteppes, specifically extensive grasslands, and rain‐fed cereal farmland (Morales et al., 2005; Silva et al., 2014). Its decline is thought to be caused mainly by the aforementioned intensification of agriculture (Bretagnolle et al., 2018; Traba & Morales, 2019), which results in low breeding rates caused by nest destruction and the decrease in food resources critical for chick survival, such as arthropods (Bretagnolle et al., 2011, 2018). Productivity of this species has been shown to be extremely low in many areas of its breeding range, including farmland areas of the Iberian Peninsula (Lapiedra et al., 2011; Morales et al., 2008), and has been considered to be insufficient for population viability (Morales et al., 2005).

Most knowledge about the relationship between farmland characteristics and food resources for the species comes from France (i.e., Bretagnolle & Inchausti, 2005; Jiguet, 2002; Salamolard & Moreau, 1999). Insufficient knowledge exists, however, in the core area of the distribution of the species in the Iberian Peninsula, where farmland is relatively less intensive, and where certain less‐productive farmland areas suffer from abandonment (rather than intensification). A better knowledge of how arthropod availability varies in farmland habitats at the critical time of chick rearing, and whether variation is related to variables that may be modified through management, such as vegetation type or structure, would be particularly important for helping design efficient management measures aimed to improve the breeding success of the species.

The aims of this study are: (a) to evaluate the trophic offer (i.e., arthropod abundance) for little bustards in different agricultural habitats during the chick‐rearing period in Mediterranean cereal farmland; (b) to examine the relationship between arthropod abundance and vegetation structure; and (c) to assess whether areas used by the species in this period differ in their arthropod abundance from nonused areas. These aims are addressed in four study sites under different agricultural management intensities, which allows assessing and discussing results in the framework of increasing agriculture intensification.

## 2 MATERIALS AND METHODS

### 2.1 Study areas

**FIGURE 2:**
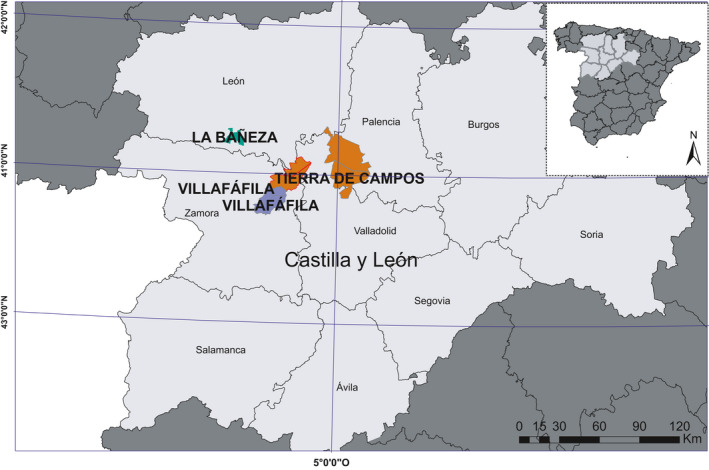
Map of the study sites. SPAs where sampling was carried out were highlighted in color (green for La Bañeza, purple for Villafáfila, and brown for Tierra de Campos)

Fieldwork was conducted in the provinces of Valladolid, Zamora, and León (Northwest Spain; Figure 2), and more specifically in four different sites: the Wildlife Reserve of Villafáfila (2 sites), Tierra de Campos (1 site), and La Bañeza (1 site). The Wildlife Reserve of Villafáfila is a protected area with an extension of 32.549 ha and an average altitude of 700 m.a.s.l. (meters above sea level), and its management is conditioned by conservation goals (protection of pseudosteppe areas and birds). It is designated as a Special Protection Area (SPA) within Spain's Natura2000 network. Nevertheless, there are differences in farmland management within the Reserve (Rodríguez Alonso & Palacios Alberti, 2006), and thus, we selected two different localities for sampling: Villafáfila North, coinciding with the northwestern half of the Reserve (less intensive; see below), and Villafáfila South, coinciding with the south‐eastern half (more intensive; see below). Tierra de Campos encompasses territories from four different SPAs: Penillanuras‐Campos Norte, Penillanuras‐Campos Sur, La Nava Campos Norte, and La Nava Campos Sur, which extend over 131,187.6 ha with an average altitude of 750 m.a.s.l., mostly devoted to intensive cereal farmland (Rodríguez‐Pastor et al., 2016). Finally, La Bañeza is located within Valdería de Jamuz SPA (9,713.2 ha, 800 m.a.s.l) and shows a marked degree of agricultural abandonment, including woodland and scrub patches interspersed with arable fields and grasslands. The four study sites are under continental Mediterranean climate with cold winters and warm summers, and rainfall was distributed mainly between October and June.

The four sites are dominated by dry cereal farmland, although there are differences between them in relation to the proportion of cultivated land (17.63% in La Bañeza, 40.26% in Villafáfila North, 39.58% in Villafáfila South, and 42.71% in Tierra de Campos) and noncultivated land (51.21% in La Bañeza, 19.45% in Villafáfila North, 25.53% in Villafáfila South, and 11.63% in Tierra de Campos; ITACYL, 2019). Average field size is 0.72 ha in La Bañeza, 0.92 ha in Villafáfila North, 1.11 ha in Villafáfila South, and 1.56 ha in Tierra de Campos. These two landscape features are recognized landscape‐level indicators of agriculture intensification (Emmerson et al., 2016; Jareño, 2014). In addition, the four study sites differ in their representation of alfalfa fields: 0.61% in La Bañeza, 19.54% in Villafáfila North, 10.86% in Villafáfila South, and 10.67% in Tierra de Campos. In Villafáfila, alfalfas are rain‐fed and mainly aimed to provide habitat for great bustards and other steppe birds. In Tierra de Campos, a large proportion of the alfalfas are irrigated (Jareño, 2014) and mown several times per year. Cereal is rain‐fed in all sites. Based on these figures, Tierra de Campos can be considered as the most intensively farmed site, followed by Villafáfila South and Villafáfila North, while La Bañeza is the least intensive one.

### 2.2 Study species

The little bustard is a sexually dimorphic bird that breeds in exploded leks (Jiguet et al., 2002). Clutch size is 3–4 eggs, which are incubated by females for 20–22 days (Cramp & Simmons, 1980; Cuscó et al., 2021). Chicks are reared only by females (Cramp & Simmons, 1980), which contributes to explain sex differences in microhabitat (i.e., vegetation structure) selection in the breeding season (Devoucoux et al., 2019; Morales et al., 2008; Silva et al., 2014). Breeding males seek conspicuousness in more open areas that also contain food resources, while females prefer fields with high cover and enough resources to breed their offspring (Morales et al., 2008). Male territories, however, tend to be located in areas with resources favored by females and families (Morales et al., 2013; Traba et al., 2008).

In the Iberian Peninsula, the breeding season spreads between mid‐April and mid‐July (Cuscó et al., 2021). Hatching occurs on average in the first half of June, although families are usually not detectable until cereal is harvested, a time when vegetation cover decreases drastically, and families begin to forage on stubble fields (Bretagnolle et al., in press; Tarjuelo et al., 2013). This usually occurs from early June to late July, depending on each locality's cereal phenology. The brood is considered successful when chicks are 30 days old (Lapiedra et al., 2011). From then on, their mortality risk decreases considerably and reach adult levels (Inchausti & Bretagnolle, 2005; Morales, Bretagnolle, et al., 2005). Chicks, however, remain with their mother in the same postbreeding flock after that time and until migration.

Adult little bustards are basically herbivorous (Bravo et al., 2017; Jiguet, 2002). However, chicks’ diet during the firsts 3 weeks of their life consists exclusively of arthropods (Cramp & Simmons, 1980). Jiguet (2002) found that Dermaptera, Coleoptera, and Orthoptera were important components of little bustard chick diet, particularly the latter two. Consequently, arthropod availability in summer when juveniles are growing is crucial for the species’ breeding success (Jiguet, 2002). In particular, orthopterans reach a peak of abundance during this period (July–August; Louveaux, 1991) and therefore become a main source of food for chicks (Jiguet, 2002).

### 2.3 Data collection

Little bustard censuses were carried out in 2019 during late May (SEO/Birdlife, 2019) and repeated again in early July (Table A1). Surveys were done by car, stopping regularly between 500 and 1,000 meters at high visibility points from which the landscape was scanned for little bustards. We aimed to detect males, females, and (in July) families or postbreeding groups (adults plus juveniles). However, females are extremely shy and hardly detectable prior to hatching (García de la Morena et al., 2018). Surveys were done during the two hours following sunrise or preceding sunset, coinciding with little bustard activity peaks (see Faria & Morales, 2018; Morales et al., 2008; Tarjuelo et al., 2013 for similar methodology). The number of stops varied between the study sites according to their extension and the potential habitat for the species. In total, 166 stops were done in Villafáfila Reserve, 53 in La Bañeza, and 151 in Tierra de Campos.

**FIGURE A1:**
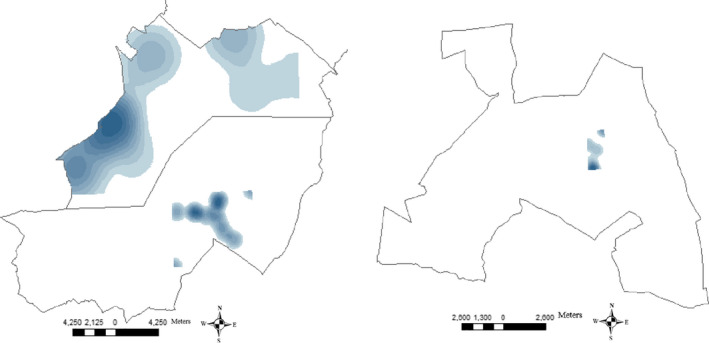
90% Kernel maps (areas used by little bustards, from observations in May, in blue) in Villafáfila North, Villafáfila South (left), and La Bañeza (right)

Based on little bustard observations during May surveys, kernel density areas were calculated to identify areas used by the species at each study site (Figure A1). Although the census methodology is particularly appropriate to detect breeding males, little bustard observations registered were overall scarce (Table A1) due to the small population size and low density in the region (García de la Morena et al., 2018). Therefore, we decided to use all observations (of both males and females) to calculate the kernel density areas. Previous studies have shown that females and families tend to occur within the areas where males are seen (Jiguet et al., 2002; Morales et al., 2013; Tarjuelo et al., 2013). Thus, in the absence of enough female‐only data, this approach renders adequate estimates of areas used by females, families, and postbreeding flocks in which families integrate. No observations were recorded in Tierra de Campos in any census. Kernel parameters were adjusted to the spatial frame and number of little bustard observations obtained at each site (La Bañeza: cell size = 3.16, smoothing factor = 426.707; Villafáfila North: cell size = 63.83, smoothing factor = 3,186.377; Villafáfila South: cell size = 21.23, smoothing factor = 913.126; and ArcGis 10.4.1 cell size and smoothing factor were calculated by default to reach a plausible result avoiding disjunct distributions). We defined as “used” the areas comprised by the 90% probability isopleth of kernel areas in order to avoid the influence of extreme fixes (Cuscó et al., 2021; Kenward et al., 2001). A similar approach has been previously used in other little bustard studies (see Jiguet et al., 2002; Tarjuelo et al., 2013; Traba et al., 2008). The used area was 80.45 ha in La Bañeza (0.8% of the whole extension of that site), 13,221.42 ha in Villafáfila North and 1,816.32 ha in Villafáfila South (53.32% and 5.37%, respectively), and the remainder was considered as “not used.” In the case of Tierra de Campos, all the area was considered as nonused since no little bustards were recorded during the censuses. Used and nonused areas considered in our analyses had similar landscape characteristics within their respective study site (i.e., none included forests, woody crops, water bodies, or other habitat types directly unsuitable for little bustards).

**FIGURE A2:**
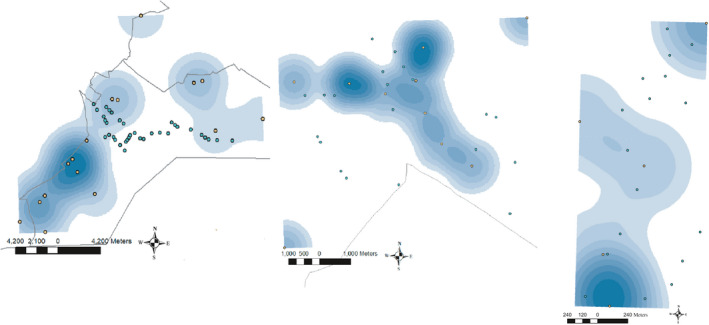
Maps with kernel density areas of Villafáfila North (left), Villafáfila South (center), and La Bañeza (right). Used areas from kernels analysis as blue, nonused in white, green points represent pitfall traps distribution, and brown points are little bustard observations from May census

**FIGURE A3:**
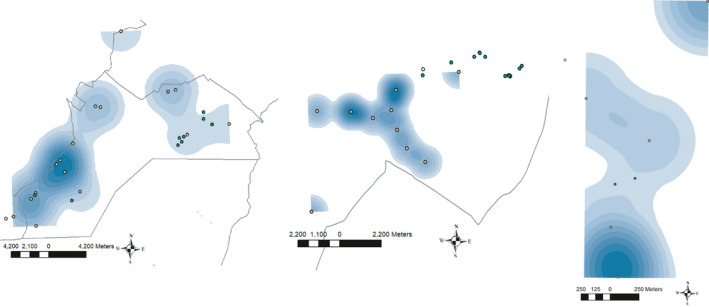
Maps with kernel density areas of Villafáfila North (left), Villafáfila South (center), and La Bañeza (right). Used areas from kernels analysis as blue, nonused in white, and points represent little bustard observations: brown ones from May, white ones from July, and green ones from groups detected during July–August censuses

Arthropods were sampled in both used and nonused areas from mid‐July to early August 2019 by means of pitfall traps of 70 mm of diameter containing a mix of 50% ethylene glycol and drops of detergent (to break surface tension). Even if, theoretically, nonused areas may include “false negatives,” the census carried out in July (Table A1) and the monitoring of postbreeding groups yielded no little bustard observations in zones classified as “nonused” based on kernel analyses and where pitfalls were disposed (Figures A2 and A3). Therefore, we consider that our sampling design was adequate to separate used and nonused areas, regardless of possible census limitations.

Each sampling point consisted in a row of three pitfall traps separated 5 m from each other and from the field margin. They were collected after a week (see Guerrero et al., 2010 for a similar procedure). At that moment, a sweep net was used along a 25‐meter transect to collect species for which the pitfall traps are unsuitable as sampling method (Capinera, 2010): We swept the air (to catch flying species) and the ground vegetation (to catch those species hidden there). Pitfall and sweep‐net samples from each point were pooled and fixed in a labeled plastic jar with 70% ethanol. Pitfalls were stratified according to the most representative habitats in the study areas: alfalfa crops, field margins, pastures, and stubbles, and their numbers were distributed according to each habitat's extension in each study site (Table 1). In a few sampling points (*N* = 8), some pitfalls were accidentally removed or trampled by livestock or machinery before collection and were thus discarded from analyses.

**TABLE 1:** Mean (± standard deviation, *SD*) abundance, biomass, and taxonomic richness estimated from pitfalls in different localities and habitats (U: used areas; N: nonused areas). Sample size indicates number of sampling points

	Sample size		Total abundance (No. of individuals)		Total biomass (mg)		Richness (No. of orders)	Orthopteran abundance (No. of individuals)		Orthopteran biomass (mg)		Coleopteran abundance (No. of individuals)		Coleopteran biomass (mg)	
	U	*N*	Mean	*SD*	Mean	*SD*		Mean	*SD*	Mean	*SD*	Mean	*SD*	Mean	*SD*
La Bañeza	7	10	264.3	273.9	2,211.3	973.3	19	11.8	7.4	1,553.4	999.5	7.5	6.7	168.7	150.8
Field margin	2	2	349.3	194.6	1,745.0	295.2	14	6.8	1.9	887.7	291.6	7.0	7.0	157.5	156.9
Grazed field	4	5	281.7	332.7	2,637.1	1,120.0	17	15.4	8.0	2,031.1	1,116.1	4.7	2.2	105.0	50.3
Stubble	1	3	99.0	79.8	1,555.4	246.5	11	7.7	0.9	1,008.2	151.9	16.7	9.0	374.9	202.9
Tierra de Campos	0	41	857.9	3,245.5	2,205.1	4,238.8	14	6.5	7.0	856.6	932.3	4.7	8.0	105.8	179.1
Alfalfa	0	10	2,273.9	6,235.7	4,566.4	7,822.0	12	10.5	10.1	1,380.8	1,396.2	4.6	6.3	103.5	140.8
Field margin	0	10	253.3	187.9	1,306.7	1,077.7	12	6.1	5.5	803.7	768.1	3.2	5.2	72.5	117.3
Grazed field	0	11	329.5	211.1	1,454.2	947.0	10	4.9	4.5	644.4	630.5	9.3	12.3	209.2	275.7
Stubble	0	10	428.8	369.1	1,203.0	550.4	11	4.0	3.1	526.0	439.0	0.8	0.7	16.9	15.9
Villafáfila North	18	17	1,341.7	3,761.9	5,155.5	3,008.0	21	24.8	13.2	3,258.9	1,768.4	31.8	25.3	715.6	568.3
Alfalfa	5	5	3,490.1	6,418.9	7,116.1	3,695.9	18	29.6	7.9	3,892.6	1,088.3	31.8	22.8	715.3	512.5
Field margin	3	3	324.3	126.9	3,609.0	1,720.2	16	19.3	12.5	2,542.5	1,800.6	24.2	25.3	543.6	568.4
Grazed field	4	3	377.3	202.2	4,464.1	2,694.0	14	25.0	15.4	3,287.7	2,216.2	25.7	27.2	577.3	612.5
Stubble	6	6	382.4	222.1	4,537.6	2,285.1	18	23.1	14.8	3,037.8	2,052.8	40.1	27.8	902.0	625.2
Villafáfila South	10	10	462.6	500.1	3,985.9	2,312.7	18	23.4	13.3	3,077.3	1,793.2	9.5	12.0	213.7	269.8
Alfalfa	3	3	519.2	382.0	5,008.5	2,092.3	13	31.5	11.9	4,142.5	1,714.1	5.5	5.6	123.7	126.2
Field margin	2	2	198.8	44.2	2,654.4	981.9	15	15.3	6.5	2,005.5	980.4	9.8	3.3	219.3	74.3
Grazed field	2	2	569.3	319.0	4,269.5	1,419.5	12	23.8	8.3	3,123.3	1,253.9	14.8	23.6	331.8	529.9
Stubble	3	3	510.8	819.0	3,662.0	3,360.8	11	20.5	16.0	2,695.9	2,303.1	9.8	11.6	221.2	260.5
Total	35	78	839.6	2,842.0	3,444.4	3,430.4	23	16.1	13.4	2,117.9	1,776.8	14.3	19.6	321.8	440.6

At the same time, we estimated vegetation structure at each sampling point using a 50 × 50 cm quadrat at the location of each pitfall; therefore, we had three measurements to characterize each sampled field. To assess horizontal structure, we visually estimated the following percentage covers inside the quadrats: (a) bare ground, (b) litter, (c) green vegetation, (d) weeds, and (e) total vegetation cover. To measure vertical structure, a ruled rod was used to record contacts at different heights (below 5 cm, between 5 and 10 cm, between 10 and 30 cm, and above 30 cm), total number of contacts, and maximum vegetation height inside the quadrat. As a measure of habitat plant diversity, we counted the number of different species in the square.

### 2.4 Arthropod identification and quantification

Each arthropod individual was visually identified to order (Barrientos, 1988; Chinery & Costa, 2006). Abundance was estimated as the total number of individuals trapped per sampling point. We use “abundance” to refer to the number of individuals trapped for simplicity, although this number is a reflection of both activity and density. Biomass was calculated using the equations developed by Hódar (1996), which require the average length of each group. For this purpose, the length of 30 individuals per order was measured, except when the sample for a particular order was <30, in which case the mean was obtained from all the sampled individuals (see Tarjuelo et al., 2019 for similar procedures). Total and per‐order values were estimated. Finally, to evaluate whether little bustards were associated with areas with higher arthropod diversity, richness was calculated as the total number of orders identified per sampling point.

### 2.5 Statistical analyses

All analyses were performed with *R software version 3.6.2* (R Core Team, 2019), and the packages are as follows: *car* (Fox & Weisberg, 2019), *MASS* (Venables & Ripley, 2002), *lsmeans* (Lenth, 2016), and *stats* (R Core Team, 2019). The graphs presented summarising the results were done with the package *ggplot2* (Wickham, 2016).

**FIGURE A4:**
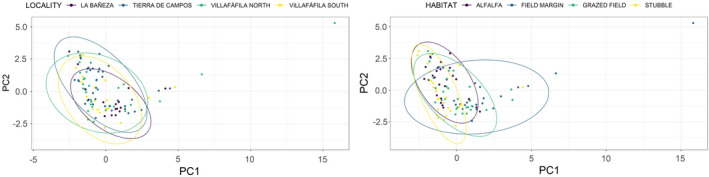
Biplot of PC1 and PC2 values. Values for each study locality (above) and habitat (below) are indicated in different colors

We computed a principal component analysis (PCA) for vegetation structure and diversity variables in order to synthesize the information. Since the vegetation variables were standardized (as the value minus the average divided by the standard deviation), we used the covariance matrix to calculate the PCA. The PCA yielded 12 components, but only the first three presented eigenvalues higher than 1 (Table 2). Among those, only PC1 and PC2 had a clear ecological interpretation and were thus included in subsequent models as explanatory variables (Table 2). PC1 showed high positive correlations with the variables that measured contacts at different heights and thus can be interpreted as variation in vegetation vertical structure; positive values of PC1 thus imply more complexity in the vertical vegetation structure. PC2 was correlated with percentage covers (negatively with those of vegetation cover, particularly with total vegetation cover, and positively with bare ground cover) and was interpreted as a gradient in vegetation cover: positive values of PC2 indicate low vegetation cover. Together, both principal components explained 62.28% of the variance (Table 2 and Figure A4).

**TABLE 2:** Summary results from PCA for the vegetation structure variables. Only the principal components with an eigenvalue higher that 1 and clear ecological interpretation are presented

Loadings	PC1	PC2
Maximum height	0.3014	0.0747
Litter cover	−0.0696	−0.4309
Weed cover	0.2756	−0.2353
Full cover	0.2371	−0.5314
Green cover	0.0155	0.1157
Contacts between 5 and 10 cm	0.3774	0.1965
Contacts between 10 and 30 cm	0.3868	0.2019
Contacts above 30 cm	0.3212	0.1912
Contacts below 5 cm	0.3355	0.0884
Species number	0.2375	−0.1687
Contacts	0.3912	0.1478
Bare ground cover	−0.2339	0.5324
Eigenvalues	5.105	2.396
Cumulative proportion of variance	42.38%	62.28%

**FIGURE A5:**
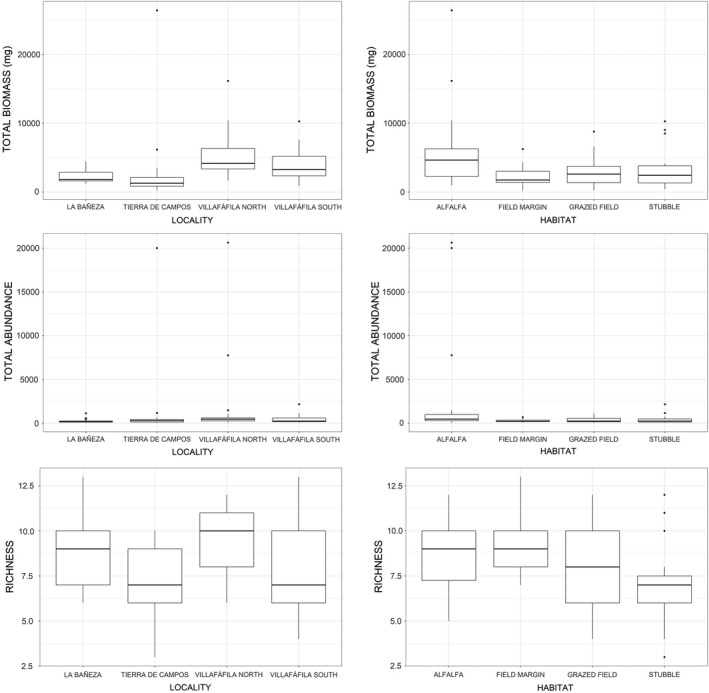
Boxplots of raw data of biomass, abundance, and richness for each locality and habitat

Exploratory plots of raw data against the main explanatory variables are shown in Figure A5. We implemented general linear models (GLMs) to analyze factors explaining variation in arthropod abundance, biomass, or richness. Abundance and biomass were, as expected, correlated, although the relationship was not linear (results not shown). Nevertheless, we kept both response variables for analyses, given their different biological meaning. GLMs for total biomass, total abundance, total richness, orthopteran biomass, and coleopteran biomass assumed Gaussian distribution of response variables, while negative binomial generalized linear models were used for orthopteran and coleopteran abundance. In the case of total biomass, total abundance, and coleopteran biomass, we used a log (*x* + 1) data transformation in order to meet normality and variance homogeneity requirements. In all models, the explanatory variables used were as follows: little bustard use (a two‐level factor: area used or not used by little bustards), habitat (with four levels: alfalfa, field margin, grazed field, and stubble), locality (four levels: La Bañeza, Tierra de Campos, Villafáfila North, and Villafáfila South), and the principal components PC1 and PC2. We also included the interaction between use and habitat to examine whether potential habitat differences were similar or not between used and nonused areas.

Model selection was carried out using the *drop1* function, which compares the deviance of the full model with the deviance of a model in which each term is dropped, testing whether the difference in deviance is significant using an *F* test (Zuur et al., 2009). We applied this function sequentially, eliminating at each step nonsignificant variables (starting with interactions). In some instances, the final step included a marginally nonsignificant variable when the model with that term yielded better residual structure, and thus higher global model fit. For each final model, the structure of residuals was checked by examining normality histograms, residual versus predicted value plots, and q‐q plots. For each final model, we present type III results for the significance of each variable (calculated with the ANOVA function in *car* package; Table 3), as well as graphical outputs of the directions and size of the effects (drawn with the *ggplot2* package). The parameter estimates and their standard errors are also specified in Table A2. The ANOVA function provides *F* tests for linear models, and chi‐square statistics for general linear models using binomial error distributions. In addition, Tukey's tests were carried out in order to assess differences between category levels (Table A3).

**TABLE 3:** Type III *F* tests (for linear models) or chi‐square tests (for negative binomial models) of the final models explaining variation in arthropod availability measured through biomass, abundance, and richness

Model No	Response variable	Explanatory variables	Degrees of freedom	Statistic	*p*	Adjusted *R* ^2^/deviance
1	Total biomass	Habitat	3	*F* = 6.878	0.0003	0.4647
		Locality	3	*F* = 23.410	1.65 e‐11	
2	Total abundance	Use	1	LR‐Chisq = 3.883	0.049	0.1391
		Habitat	3	LR‐Chisq = 17.188	0.0006	
3	Richness	Habitat	3	LR‐Chisq = 31.616	6.304 e‐7	0.3479
		Locality	3	LR‐Chisq = 34.217	1.783 e‐7	
4	Orthopterans biomass	Use	1	*F* = 15.431	0.012	0.4419
		Habitat	3	*F* = 6.534	0.010	
		Locality	3	*F* = 13.340	2.323 e‐7	
5	Orthopterans abundance	Use	1	LR‐Chisq = 5.211	0.022	0.513
		Habitat	3	LR‐Chisq = 16.876	0.0007	
		Locality	3	LR‐Chisq = 63.010	1.337 e‐13	
6	Coleopterans biomass	Use	1	*F* = 2.530	0.12	0.3832
		Habitat	3	*F* = 2.020	0.12	
		Locality	3	*F* = 20.297	3.345 e‐10	
		PC1	1	*F* = 4.989	0.028	
		Use*Habitat	3	*F* = 2.600	0.057	
7	Coleopterans abundance	Use	1	LR‐Chisq = 0.781	0.38	0.463
		Habitat	3	LR‐Chisq = 9.958	0.019	
		Locality	3	LR‐Chisq = 83.063	<2 e‐16	
		PC1	1	LR‐Chisq = 6.078	0.014	
		Use*Habitat	3	LR‐Chisq = 11.322	0.010	

## 3 RESULTS

Twenty‐three arthropod orders were identified. The most abundant were collembolans, hymenopterans, orthopterans, and coleopterans. Values per study site and habitat of the variables analyzed are presented in Table 1. Villafáfila North yielded the highest values for all variables (Table 1), followed by Villafáfila South in the case of total biomass, and orthopteran and coleopteran biomass and abundance, and by Tierra de Campos in the case of total abundance. The highest richness values were found in Villafáfila North, followed by La Bañeza, Villafáfila South, and Tierra de Campos. Alfalfa crops presented the highest values of all variables in all sites where they were sampled, except for coleopterans, whose abundance and biomass tended to be higher in pastures and stubbles (Table 1).

**FIGURE 3:**
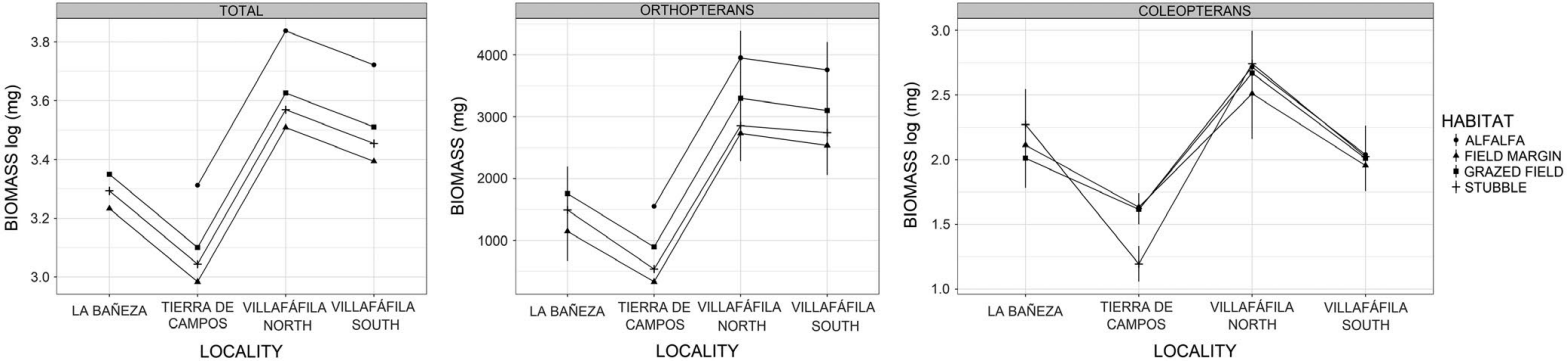
Mean (± *SD*) predicted total, orthopteran, and coleopteran biomass (mg) across the study localities and habitats (based on parameter estimates of models 1, 4, and 6 in Table 3). Note that total and coleopteran biomass data were log (*x* + 1)‐transformed, so they should be represented at different scales

**FIGURE 4:**
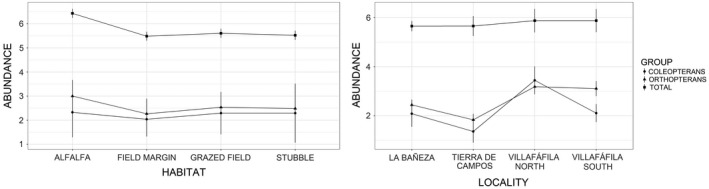
Mean (± *SD*) predicted total (log (*x* + 1) transformed), coleopteran, and orthopteran abundance across the study sites and habitats (based on parameter estimates of models 2, 5, and 7 in Table 3)

**FIGURE 5:**
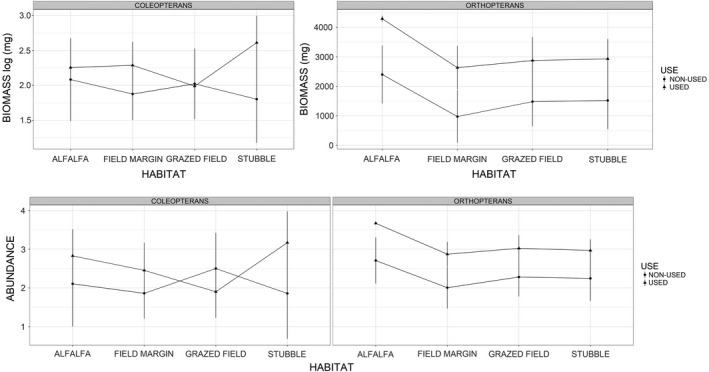
Mean (± *SD*) predicted coleopteran and orthopteran biomass (mg) and abundance across habitats and used/nonused areas by little bustards (based on parameter estimates of models 4, 5, 6, and 7 in Table 3). Coleopteran biomass data were log (*x* + 1)‐transformed; so, to gain a better understanding, each taxon was plotted separately according to their scale range

According to the GLMs (Table 3), total arthropod biomass varied significantly between localities and habitats, with highest levels recorded in Villafáfila North and alfalfa fields, respectively, and lowest levels in Tierra de Campos and field margins (parameter estimates from final models presented in Table A2, results shown graphically in Figure 3). On the other hand, total abundance varied with habitat and little bustard use (Table 3), being higher in alfalfa fields than in other habitats (Table A2, Figure 4), and in areas used by little bustards compared with nonused areas (Table A2, Figure 5).

**FIGURE 6:**
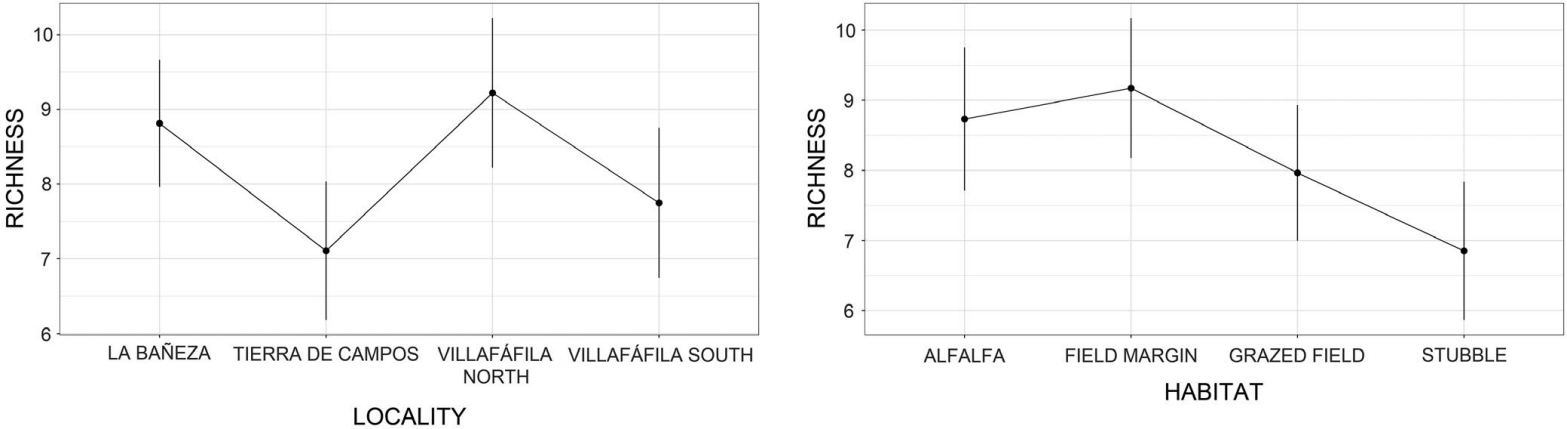
Mean (± *SD*) predicted arthropod richness across study sites and habitats (based on parameter estimates of model 3 in Table 3)

Variation in arthropod richness was also associated with habitat and locality (Table 3). However, the highest richness values were found in field margins, while the significance of locality was due to the low values of Tierra de Campos (Table A2, Figure 6).

**FIGURE 7:**
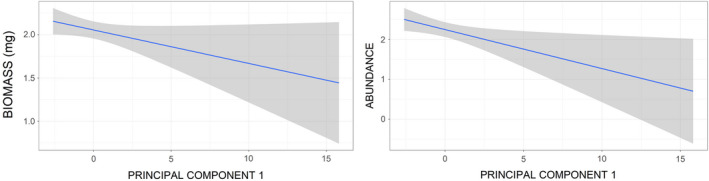
Predicted effect of vegetation vertical complexity (PC1) on coleopteran biomass and abundance (based on parameter estimates of models 6 and 7 in Table 3)

In the case of orthopterans, both biomass and abundance showed similar results, being significantly influenced by little bustard use, habitat, and locality (Table 3). For coleopterans, only locality and PC1 had a significant effect on biomass, while abundance was related to locality, PC1, and the interaction of little bustard use with habitat (Table 3). The latter arose because coleopteran abundance was higher in used areas for all habitats specially in stubbles; however, coleopteran abundance was higher in nonused than in used grazed fields (Table A2, Figure 5). Coleopteran abundance and biomass were higher in plots with lower PC1 values (Table A2, Figure 7).

## 4 DISCUSSION

Our results show that although field margins were the taxonomically richest habitat, alfalfa fields presented significantly higher total arthropod biomass and abundance (including orthopterans) than other dominant habitats in the study localities (stubbles or pastures). Arthropod abundance (including orthopteran and coleopteran abundance and biomass) varied also significantly between localities, and accordingly with the management intensification gradient. Areas used by little bustards had higher orthopteran and coleopteran abundance and biomass than nonused areas, except for grazed fields where coleopteran abundance was higher in nonused than in used ones.

Little bustard chicks and fledglings are almost exclusively insectivorous during their 2–3 first weeks of life (Jiguet, 2002). More specifically, coleopterans and orthopterans have been shown to play a key role in little bustard juvenile diet (Jiguet, 2002): Large ground beetles predominate, but grasshoppers gain importance as chicks grow. Moreover, little bustard chicks need on average 200 grasshoppers per day to complete their growth, adjusting prey size to their own age and size (Bretagnolle et al., 2021). Not surprisingly, the availability of both coleopterans and orthopterans seems to be determinant in the space and habitat use of little bustard males and families (Badenhausser et al., 2009; Bretagnolle et al., 2011; Traba et al., 2008). The significantly higher orthopteran abundance and biomass in little bustard‐used compared with nonused areas found in this study, as well as the higher coleopteran abundance found in used stubbles (see also below), are consistent with these findings. These results emphasize the influence of orthopterans and coleopterans in little bustard space use during the breeding season and highlight that breeding habitat quality for the little bustard is strongly linked to these insect orders. These results also highlight the importance of managing grasslands (including dry alfalfa fields, see below) so they can provide this key food resource for growing little bustard juveniles and females, particularly in populations where productivity and female survival are low. Productivity has been shown to be far from the threshold required for population viability (1 chick/ female and year; Morales, Bretagnolle, et al., 2005) in different Spanish populations of the species under intensive agricultural management, where it ranged from 0.27 to 0.4 chicks/ female and year (Lapiedra et al., 2011; Morales et al., 2008), while female survival is suspected to be low in most populations of western Europe, judging from their male‐biased sex ratios (Serrano‐Davies et al., unpublished data).

Except for total abundance, all arthropod‐availability variables showed significant differences between localities, with Villafáfila North (the most extensively managed locality) showing the highest values and Tierra de Campos (the most intensive of our study sites) the lowest. Coleopterans have been shown to decline with intensive agricultural management, especially large ground beetles that are more sensitive to intensification than small species (Magura et al., 2006; Postma‐Blaauw et al., 2010). Consistently, we found that coleopteran abundance was significantly lower in Tierra de Campos, the most intensive of our study sites, than in any other locality. On the other hand, orthopteran abundance and richness increase with extensive agricultural management (Gardiner, 2006), likely favored by more complex vegetation structure in extensive crops (Gardiner et al., 2002). Marini et al., (2008) found that only few orthopteran species survive in intensive meadows, which they reach when dispersing from surrounding areas. Further, fertilizers have an indirect negative effect on orthopterans because they favor dense and homogeneous crops, whereas orthopterans prefer an open and heterogeneous vegetation structure that provides a variety of microhabitats (Marini et al., 2008) covering all their life cycle requirements (Willott & Hassall, 1998). According to that, vegetation encroachment following agriculture abandonment is not expected to favor orthopteran abundance either (e.g., Fartmann et al., 2012; Uchida & Ushimaru, 2014). This is also consistent with our results, which yielded significantly higher orthopteran abundance and biomass in Villafáfila (North and South), where management is focused on steppe bird conservation, than both in the highly intensive Tierra de Campos and the largely abandoned (and partly encroached) La Bañeza. In this context, we can consider Tierra de Campos the study locality with lowest habitat quality for the little bustard, which may partially explain the disappearance of breeding birds from this intensively managed area (García de la Morena et al., 2018; SEO/Birdlife, 2019, own unpublished data), while the highest breeding habitat quality would be found in the Reserve of Villafáfila. Altogether, these results corroborate the idea that both agriculture intensification and abandonment lead to the loss of habitat quality for little bustards and other steppe birds that have been shown to depend on large‐sized insects for breeding such as the great bustard (*Otis tarda*; Lane et al., 1999; Rocha et al., 2005), the lesser kestrel (*Falco naumanni*; Rocha, 1998; Lepley et al., 2000), or Montagu's harrier (*Circus pygargus*; Arroyo, 1997; García & Arroyo, 2005).

Habitat differences in arthropod availability were driven by the major role of alfalfa fields, which showed significantly higher total arthropod, as well as orthopteran, abundance, and biomass than any of the other habitats considered. Previous studies have pointed out the structure complexity of alfalfa fields, which results in high values of arthropod richness (Pearson et al., 2008; Pimentel & Wheeler, 1973). This may relate not only to the amount of arthropod food resources found in alfalfas (Forister, 2009), but also to the habitat stability provided by these multiannual crops (Summers, 1998). Therefore, numerous herbivorous insects occupy alfalfas attracting their natural enemies (Holland, 2002), including little bustard families. However, total taxonomic richness was found to be higher in field margins, which concurs with other studies (e. g. Smith et al., 2008; Woodcock et al., 2008) and may also be associated with their stability as permanent habitat (Pfiffner & Luka, 2000). Further, the natural diversity of plants found in field margins provides food resources for a wide range of insects including coleopterans and orthopterans (Smith et al., 1994). Coleopteran abundance variation in areas used by little bustards compared with nonused areas varied between habitats. While stubbles yielded significantly lower values than other habitats (with highest ones in pastures; see Figure 5) in nonused areas, in used areas they presented significantly higher coleopteran abundance than any other habitat. This result underlines the importance of stubbles as foraging habitat for little bustard families pointed out in previous studies (Tarjuelo et al., 2013): Little bustard families select stubbles probably because their simplified vegetation structure makes prey spotting and chasing easier, even if predation risk might be higher in this habitat (Lapiedra et al., 2011). The negative correlation of coleopteran biomass with vertical vegetation complexity (Figure 7) was a somehow unexpected result. However, it may reflect that in dense vegetation large ground beetles are scarce, because they do not find food (in the case of Scarabaeidae; Cole et al., 2002) and / or because many coleopterans are canopy dwellers, and thus, their probability to fall in traps is smaller. Moreover, some large‐sized coleopteran groups such as darkling beetles (Tenebrionidae) are linked to arid or semiarid environment and tend to avoid high vegetation cover (Doyen & Tschinkel, 1974). In any case, this result provides further support to the idea that little bustard families can access this relevant prey more easily in open and simple habitats such as stubbles, as shown for other farmland bird species (Whittingham & Evans, 2004).

### 4.1 Conclusions and conservation implications

Our results highlight (a) the relevance of arthropods, particularly orthopterans and coleopterans in little bustard space use during the breeding and chick‐rearing season, (b) the importance of dry alfalfa fields as food resource reservoirs for the species in this critical time of year, (c) the likely food depletion in study sites outside the Reserve of Villafáfila, and particularly the intensive farmland of Tierra de Campos, and (d) the role of stubbles as providers of an important food resource (coleopterans) during the chick‐rearing season in areas used by the species. These results are consistent with previous findings regarding the importance of agricultural management for key insect groups such as orthopterans (Bonari et al., 2017), the role of coleopterans and orthopterans in little bustard growth and habitat selection (Bretagnolle et al., 2011; Jiguet, 2002; Traba et al., 2008), and the relevance of stubbles as foraging habitat for chicks (Tarjuelo et al., 2013). We conclude that an adequate management of alfalfa fields and stubbles to provide their key food resources during this phenological phase is required to improve little breeding success and recruitment (and thus reverse the species’ decline). Measures focused in those habitats may need to be preferentially implemented in more intensively managed areas, or even in those undergoing agricultural abandonment and vegetation encroachment.

## CONFLICT OF INTEREST

The authors declare that there is no conflict of interest.

## AUTHOR CONTRIBUTIONS

**David González del Portillo:** Data curation (equal); Formal analysis (lead); Investigation (lead); Visualization (lead); Writing‐original draft (lead). **Beatriz Arroyo:** Conceptualization (equal); Funding acquisition (equal); Project administration (equal); Resources (equal); Supervision (equal); Validation (equal); Writing‐review & editing (equal). **Guillermo García Simón:** Data curation (equal). **Manuel B. Morales:** Conceptualization (equal); Funding acquisition (equal); Project administration (equal); Resources (equal); Supervision (equal); Validation (equal); Writing‐review & editing (equal).

## Data Availability

The dataset generated and analyzed during the current study is available in the CSIC Digital Repository: http://dx.doi.org/10.20350/digitalCSIC/13680

## 1

**TABLE A1:** Little bustard observations during May and July census

Study site	Date	Total individuals	Males	Females like	Undetermined
Villafáfila South	16/05/2019	8	8	0	0
Villafáfila South	17/05/2019	4	4	0	0
Villafáfila South	09/07/2019	4	2	1	1
Villafáfila North	13/05/2019	7	7	0	0
Villafáfila North	15/05/2019	2	2	0	0
Villafáfila North	20/05/2019	1	1	0	0
Villafáfila North	25/05/2019	2	2	0	0
Villafáfila North	29/05/2019	2	2	0	0
Villafáfila North	30/05/2019	2	2	0	0
Villafáfila North	01/06/2019	3	2	1	0
Villafáfila North	10/07/2019	5	0	0	5
La Bañeza	18/05/2019	5	5	0	0
La Bañeza	23/05/2019	4	3	1	0
La Bañeza	11/07/2019	9	1	0	8

**TABLE A2:** Parameter estimates from models described in Table 3 and represented in the Figures 3, 4, 5, 6, 7

Response variable	Explanatory variable	Estimate	*SE*	*T* value	Pr (>*t*)
Total biomass	Intercept	3.561	0.094	38.089	<2e^‐16
	Field margin	−0.328	0.078	−4.191	6.09e^‐5
	Grazed field	−0.211	0.077	−2.753	0.007
	Stubble	−0.268	0.074	−3.597	0.0005
	Tierra de Campos	−0.249	0.083	−2.988	0.003
	Villafáfila North	0.276	0.087	3.180	0.002
	Villafáfila South	0.161	0.094	1.711	0.090
Total abundance	Intercept	6.314	0.199	31.720	<2e^‐16
	Used	0.397	0.201	1.971	0.051
	Field margin	−0.947	0.276	−3.429	0.0008
	Grazed field	−0.844	0.261	−3.237	0.002
	Stubble	−0.921	0.265	−3.473	0.0007
Richness	Intercept	9.584	0.593	16.162	<2e^‐16
	Field margin	0.436	0.497	0.878	0.382
	Grazed field	−0.882	0.487	−1.812	0.073
	Stubble	−2.051	0.472	−4.345	3.4e^‐5
	Tierra de Campos	−1.900	0.529	−3.595	0.0005
	Villafáfila North	0.359	0.551	0.652	0.516
	Villafáfila South	−1.130	0.596	−1.895	0.061
Orthopteran biomass	Intercept	1,954	427.4	3.928	0.0001
	Used	825.5	322.9	2.556	0.012
	Field margin	−1,222.4	386.5	−3.162	0.002
	Grazed field	−656.2	378.6	−1.733	0.086
	Stubble	−1,014.4	367.4	−2.761	0.006
	Tierra de Campos	−403.4	449.8	−0.897	0.372
	Villafáfila North	1,587.2	429.7	3.694	0.0003
	Villafáfila South	1,390.6	464.3	2.995	0.003
Orthopteran abundance	Intercept	2.703	0.222	12.151	<2e^‐16
	Used	0.318	0.139	2.286	0.022
	Field margin	−0.6125	0.1745	−3.511	0.0004
	Grazed field	−0.321	0.168	−1.914	0.055
	Stubble	−0.555	0.163	−3.404	0.0006
	Tierra de Campos	−0.513	0.209	−2.457	0.014
	Villafáfila North	0.681	0.191	3.559	0.0003
	Villafáfila South	0.599	0.206	2.912	0.004
Coleopteran biomass	Intercept	2.276	0.234	9.748	6.99e^‐16
	Used	−0.435	0.274	−1.591	0.115
	Field margin	0.048	0.229	0.208	0.836
	Grazed field	0.020	0.204	0.099	0.922
	Stubble	−0.403	0.204	−1.975	0.051
	Tierra de Campos	−0.689	0.209	−3.303	0.001
	Villafáfila North	0.605	0.200	3.031	0.003
	Villafáfila South	−0.096	0.215	−0.445	0.657
	PC1	−0.069	0.031	−2.234	0.028
	Used*Field margin	0.227	0.380	0.596	0.553
	Used*Grazed field	−0.024	0.357	−0.068	0.946
	Used*Stubble	0.837	0.361	2.319	0.023
Coleopteran abundance	Intercept	2.183	0.368	5.924	3.13e^‐9
	Used	−0.395	0.422	−0.937	0.349
	Field margin	0.328	0.374	0.879	0.380
	Grazed field	0.611	0.325	1.880	0.060
	Stubble	−0.463	0.334	−1.387	0.165
	Tierra de Campos	−0.993	0.332	−2.994	0.003
	Villafáfila North	1.411	0.309	4.559	5.13e^‐6
	Villafáfila South	0.003	0.338	0.009	0.993
	PC1	−0.153	0.051	−2.984	0.003
	Used*Field margin	−0.134	0.595	−0.225	0.822
	Used*Grazed field	−0.899	0.558	−1.611	0.107
	Used*Stubble	0.998	0.562	1.775	0.076

**TABLE A3:** Results from the Tukey post hoc comparisons. Groups are made according to the significant differences among levels (same letter indicates no significant difference between levels)

Model	Explanatory variable	Levels	Groups
Total biomass	Habitat	Alfalfa	A
		Field margin	B
		Grazed field	B
		Stubble	B
	Locality	La Bañeza	A
		Tierra de Campos	B
		Villafáfila North	C
		Villafáfila South	AC
Total abundance	Use	Used	A
		Nonused	B
	Habitat	Alfalfa	A
		Field margin	B
		Grazed field	B
		Stubble	B
Richness	Habitat	Alfalfa	AB
		Field margin	B
		Grazed field	AC
		Stubble	C
	Locality	La Bañeza	A B
		Tierra de Campos	C
		Villafáfila North	B
		Villafáfila South	AC
Orthopteran biomass	Use	Use	A
		Nonused	A
	Habitat	Alfalfa	A
		Field margin	A
		Grazed field	A
		Stubble	A
	Locality	La Bañeza	A
		Tierra de Campos	B
		Villafáfila North	C
		Villafáfila South	A
Orthopteran abundance	Use	Use	A
		Nonused	B
	Habitat	Alfalfa	A
		Field margin	B
		Grazed field	AB
		Stubble	B
	Locality	La Bañeza	A
		Tierra de Campos	A
		Villafáfila North	B
		Villafáfila South	B
Coleopteran biomass	Locality	La Bañeza	A
		Tierra de Campos	B
		Villafáfila North	C
		Villafáfila South	A
Coleopteran abundance	Habitat	Alfalfa	A
		Field margin	A
		Grazed field	A
		Stubble	A
	Locality	La Bañeza	A
		Tierra de Campos	B
		Villafáfila North	C
		Villafáfila South	A
	Use*Habitat	Use Grazed field	A
		Nonused Stubble	A
		Use Alfalfa	AB
		Used Field margin	AB
		Nonused Alfalfa	AB
		Used Stubble	AB
		Nonused Field margin	AB
		Nonused Grazed field	B
